# Machine Learning-Based Methods for Enhancement of UAV-NOMA and D2D Cooperative Networks

**DOI:** 10.3390/s23063014

**Published:** 2023-03-10

**Authors:** Lefteris Tsipi, Michail Karavolos, Petros S. Bithas, Demosthenes Vouyioukas

**Affiliations:** 1 Department of Information and Communication Systems Engineering, School of Engineering, University of the Aegean, 83200 Samos, Greece; 2 Department of Digital Industry Technologies, National and Kapodistrian University of Athens, Thesi Skliro, 34400 Evia, Greece

**Keywords:** machine learning, UAV placement, artificial neural network (ANN), deep neural network (DNN), NOMA, cooperative communications, D2D

## Abstract

The cooperative aerial and device-to-device (D2D) networks employing non-orthogonal multiple access (NOMA) are expected to play an essential role in next-generation wireless networks. Moreover, machine learning (ML) techniques, such as artificial neural networks (ANN), can significantly enhance network performance and efficiency in fifth-generation (5G) wireless networks and beyond. This paper studies an ANN-based unmanned aerial vehicle (UAV) placement scheme to enhance an integrated UAV-D2D NOMA cooperative network.The proposed placement scheme selection (PSS) method for integrating the UAV into the cooperative network combines supervised and unsupervised ML techniques. Specifically, a supervised classification approach is employed utilizing a two-hidden layered ANN with 63 neurons evenly distributed among the layers. The output class of the ANN is utilized to determine the appropriate unsupervised learning method—either k-means or k-medoids—to be employed. This specific ANN layout has been observed to exhibit an accuracy of 94.12%, the highest accuracy among the ANN models evaluated, making it highly recommended for accurate PSS predictions in urban locations. Furthermore, the proposed cooperative scheme allows pairs of users to be simultaneously served through NOMA from the UAV, which acts as an aerial base station. At the same time, the D2D cooperative transmission for each NOMA pair is activated to improve the overall communication quality. Comparisons with conventional orthogonal multiple access (OMA) and alternative unsupervised machine-learning based-UAV-D2D NOMA cooperative networks show that significant sum rate and spectral efficiency gains can be harvested through the proposed method under varying D2D bandwidth allocations.

## 1. Introduction

Undoubtedly, the utilization of unmanned aerial vehicles (UAVs) as UAV flying base stations (UFBSs) is of potential interest in the context of new-generation wireless communication systems. UAV-enabled wireless communication systems can provide wireless coverage extension, capacity enhancement, communication restoration during disaster events, and aerial data collection within the framework of Internet of Things (IoT) applications [1,2]. In contrast to conventional wireless communication systems that depend on fixed terrestrial infrastructures, UFBSs are dynamic and simple to deploy and reconfigure. Thus, their use introduces several degrees of freedom in terms of flexibility, wide coverage, and communication restoration during a disaster and temporary events. However, the anticipated advantages of deploying UFBSs are heavily contingent on their precise location within the region of interest to offer terrestrial users reliable and high-quality communication [3].

### 1.1. Background

Identifying the proper horizontal and vertical locations of UFBSs concerning other ground or flying objects is one of the most challenging parts of establishing UAV-based communication systems that achieve optimal or near-optimal performance. Hence, several research attempts in the technical literature have proposed various UFBSs placement techniques to maximize the aerial network communication performance and exploit the advantages provided [4,5,6,7,8,9,10]. The authors in [4] proposed a low-complexity method that optimizes UAVs’ 2D location, admission control, and power allocation using penalty function and successive convex approximation techniques. This approach maximizes the quality of service for terrestrial users and is effective, as confirmed by simulation results. Furthermore, the authors in [5] jointly optimized the 2D locations and the transmit power of multiple UFBSs to maximize the system sum rate using a distributed learning method that achieves stochastic stability. Collisions between the UFBSs were prevented by determining their respective heights in advance. Moreover, in [6], the 2D placement and the power allocation of the UFBSs are jointly optimized to increase the UAV network’s performance. The proposed method consists of two sub-processes. The first sub-process finds the optimal 2D position, while the second further determines the optimal power allocation to maximize the terrestrial users’ total sum rate.

The works [4,5,6] have presented conventional optimization methods to determine the optimal location of the UFBSs. Notwithstanding, other approaches focus on leveraging the machine learning (ML) advantages to deal with the UFBSs placement problem [7,8,9,10]. More specifically, the authors in [7] suggested a UAV-aided offloading approach for terrestrial networks that uses an unsupervised ML method to optimize UFBS deployment in high-traffic areas. The proposed method is divided into two sub-processes—user clustering employing the k-medoids algorithm and cluster selection scheme for identifying the UFBSs with the highest offloading factor. Another ML-based solution that aims to offload terrestrial base stations (TBSs) is proposed in [8]. The proposed scheme is based on the weighted expectation–maximization algorithm and estimates both the user distribution and the downlink traffic demand to determine the optimal UFBSs location. Similarly, the authors of [9] studied the joint 3D placement and UAV-user associations in UAV-assisted networks. For the 2D positioning of UAVs, a modified version of the k-means algorithm is utilized, while for the altitude optimization problem, they propose a game theoretic approach. Simulation results have shown that the proposed scheme outperforms other trivial cases where users are associated, over iterations, with the closest UAV. Lastly, in [10], UFBSs are treated as long-term evolution (LTE)-advanced heterogeneous networks (HetNet) to cover safety incidents. In this approach, the UFBSs are deterministically positioned on a precalculated hexagonal grid with fixed placement points, restricting the placement optimality.

UFBS optimal placement increases the possibility of obtaining LoS conditions, thus enhancing the physical communication link quality. Therefore, effective resource management techniques should be utilized to optimally exploit the improved physical links and provide highly spectral efficient communication to several ground users. Towards the goal of intelligent integration of the UFBSs into fifth-generation (5G), beyond 5G (B5G), and sixth-generation (6G) communication systems, non-orthogonal multiple access (NOMA) is expected to be a fundamental radio access technique [11,12]. The basic principle of NOMA is to serve multiple users simultaneously in a single resource block (space/time/frequency) by multiplexing them in the power domain. To accomplish this objective, superposition coding (SC) is performed at the transmitter and successive interference cancellation (SIC) at the receiver [13]. Moreover, combining NOMA with high spectral efficient multiple-input multiple-output (MIMO) techniques, such as quadrature spatial modulation (QSM) [14,15], can further enhance the spectral efficiency and increase the capacity of non-terrestrial wireless networks [3].

Recent research attempts have investigated the use of NOMA to enhance the performance of UAV-enabled communication systems [16,17,18]. The authors of [16] have studied a NOMA-based UAV-enabled communication network. Specifically, a path-following algorithm is proposed to solve the max-min rate optimization problem, which is subjected to the constraints of the total power, available bandwidth, UAV altitude, and antenna beamwidth. The numerical results have shown that the NOMA scheme outperforms OMA, in terms of achievable rate, for different system parameters. Subsequently, the authors in [17] developed a novel NOMA UAV-assisted offloading architecture for cellular networks to significantly enhance the system’s spectrum efficiency. Specifically, the 3D trajectory design and power allocation optimization problem are formulated to maximize the system sum rate. For this purpose, ML-based methods, namely k-means and mutual deep Q-network (MDQN), are utilized to deal with this problem. Another strategy [18] proposes a resource allocation scheme for a UAV-assisted full-duplex (FD) NOMA system to improve spectrum efficiency, reduce terrestrial users’ power requirements, and maintain quality of service (QoS) requirements. The method utilizes a joint uplink/downlink stepwise optimization approach to solve the NP-hard optimization problem. Simulation results demonstrate that the proposed method outperforms other methods in terms of spectrum and energy efficiency.

Besides the optimal placement of the UFBS and the selection of an efficient radio access technique, leveraging physical transmission techniques can further enhance the overall UAV communication quality. Device-to-device (D2D) communication is one such technique. For instance, in highly dense urban areas where several devices coexist within a distance of a few meters, they can benefit through the utilization of a cooperative transmission scheme. Consequently, integrating D2D communications into UAV networks has recently attracted a lot of attention, and related issues have also been studied in the literature [19,20,21,22,23]. In [19], the authors have derived the closed-form expressions for the outage probability in a UAV-assisted NOMA network with D2D communication capabilities. Also, they have formulated a power control optimization problem to maximize the D2D sum rate while ensuring a minimum rate for each UAV-connected user. The proposed method is computationally efficient but has a lower sum rate than other methods, as this has been confirmed via the simulation results. Furthermore, the energy-efficient resource allocation problem in D2D communications underlying UAV-enabled networks is investigated in [20]. Especially, this study attempts to optimize the overall energy efficiency of all D2D pairs while ensuring the secrecy rates of all users via combined power control and channel allocation. Accordingly, the Lagrangian dual and Kuhn–Munkres algorithms are utilized to solve this problem. The simulation results have shown that the proposed approach performs better than other benchmark methods. Moreover, the authors of [21] exploited the advantages that UAV-assisted communications offer and effectively combined with the NOMA technique. Particularly, they present a D2D-enhanced UAV-NOMA network architecture in which D2D is added to improve the dispatching efficiency of files. So, a graph-based file dispatching protocol is provided to decrease the UAV-assisted file dispatching mission time and control interference. Simulation results confirm the benefits of the proposed D2D-enhanced UAV NOMA network architecture and the efficacy of the planned protocol. The research presented in [22] proposed a novel approach to address disaster management issues utilizing a UAV-assisted SWIPT-enabled NOMA-based D2D network. They formulated a nonlinear power allocation optimization problem that maximized the system’s energy efficiency performance and solved it using the Dinkelbach approach. Simulation results show that the advanced NOMA system outperforms the ordinary NOMA scheme. Alternately, ref. [23] has investigated a sequential optimization problem for resource allocation and communication mode selection in a UAV-assisted D2D cellular network to improve energy efficiency and ensure satisfactory transmission rates for all ground UEs. They proposed a reinforcement learning-based scheme to solve this problem, which has been shown to be effective through simulated results.

### 1.2. Contributions

As presented in the previously detailed literature review, several studies on standalone UAV networks utilize unsupervised machine learning methods such as k-means and k-medoids to place the UAV in the region of interest. However, applying these algorithms individually to a UAV-NOMA and D2D cooperative network might degrade the overall network quality while rendering the D2D network unnecessary. Hence, to achieve enhanced network quality, it is vital to consider the interactions and trade-offs between the two algorithms and the network elements and adopt an integrated approach [7,24].

Concerning the operation of the two placement methods, both k-means and k-medoids are centroid-based clustering techniques. The two methods are fed with the terrestrial users’ coordinates as an input feature to find the point where the UFBS should be placed. In such scenarios, k-means behaves well when the terrestrial users form spherical clusters without outliers [24]. In contrast, k-medoids is robust to the outliers and correctly represents the cluster center [7]. Hence, by efficiently combining k-means and k-medoids algorithms, the UAV can be positioned in the most suitable location to ensure effective coverage for D2D communication. This combined approach considers both the similarities in the data points as well as the actual data points themselves and potential outliers or noise in the data. As a result, it leads to a more precise and reliable UAV placement. Thus conspicuously, the combination of these two algorithms exploits the strengths of both k-means and k-medoids in determining the ideal UAV placement [25].

Nevertheless, whenever the UFBS needs to be relocated, it is necessary to determine the most suitable placement method by comparing the results obtained from both clustering algorithms, i.e., k-means and k-medoids. This decision-making process requires the real-time execution of both ML methods, thus increasing the overall time complexity. Also, k-means and k-medoids are clustering algorithms that can be used to group data points together based on their similarities. However, deciding which algorithm to use can be complex and may depend on several factors. Essentially, when the dataset contains non-spherical clusters, outliers, or clusters of different sizes, it is difficult to model a decision-making approach with a simple threshold boundary. Hence, this can make it challenging to identify the unsupervised ML method that should be utilized.

Inspired by this observation, the placement scheme selection (PSS) can be regarded as a supervised classification problem, which can be handled through a fully connected artificial neural network (ANN) to enhance the overall system QoS. ANNs can be used to predict which clustering algorithm to use between k-means and k-medoids because they are able to learn the underlying patterns in the data and identify which algorithm is better suited for the given dataset. Moreover, ANNs can capture complex relationships between the input data and the output cluster labels, which can be difficult to model with a simple threshold boundary.  Consequently, this paper presents and analyzes an ANN-based UAV placement scheme to enhance the network performance of an integrated UAV-NOMA and D2D cooperative network. The proposed method intelligently integrates the UFBS into the cooperative network by efficiently combining the k-means and k-medoids unsupervised ML algorithms. Concerning the UAV-NOMA and D2D cooperative network, pairs of users are simultaneously served through the UFBS, which utilizes a NOMA optimal user pairing and power allocation strategy. At the same time, terrestrial cooperation is enabled by adopting the D2D communication paradigm, thus improving the overall communication quality. To the authors’ knowledge, this is the first time supervised machine learning techniques, such as the ANN, and unsupervised machine learning algorithms, such as k-means and k-medoids, are combined to improve the integrated UAV-NOMA D2D cooperative network. Specifically, the following major contributions are provided:

- An ANN-based UFBS placement framework is established in order to improve the overall communication quality of a UAV-NOMA and D2D cooperative network. Towards this end, supervised ML algorithms (ANN) and unsupervised ML algorithms (k-means and k-medoids) are combined.
- State-of-the-art data mining strategies are presented to transform raw data into an intelligible format for ANN algorithms and avoid underfitting and overfitting drawbacks. To the best of our knowledge, it is the first time that specific strategies have been provided in the field of UAV-NOMA and D2D cooperative networks.
- A step-by-step approach on how to handle the issue of hyperparameter tuning in ANN models is provided to enhance the predictability of the UFBS placement procedure.
- For the UFBS NOMA transmission, an optimal power allocation and user pairing strategy is considered [26]. Also, the proposed scheme promotes the cooperation between aerial and D2D networks.

### 1.3. Structure

The remainder of this paper is organized as follows. Section 2 presents the considered system model, while Section 3 outlines the unsupervised machine-learning-based methods for the UFBS placement procedure. Next, the data collection, data pre-processing, learning, validation, and testing procedures, and the performance metrics of the proposed ANN-based placement scheme selection are outlined in Section 4. Finally, simulation results are given in Section 5, followed by conclusions and future directions in Section 6.

## 2. System Model

From the system point of view, we consider a cooperative UAV and D2D-aided wireless communication system, where the UFBS is mainly responsible for communication. The D2D scheme is employed between the ground mobile terminals (GMTs) to achieve higher data rates and spectral efficiency without the involvement of any additional terrestrial or flying base station.

**Figure 1:**
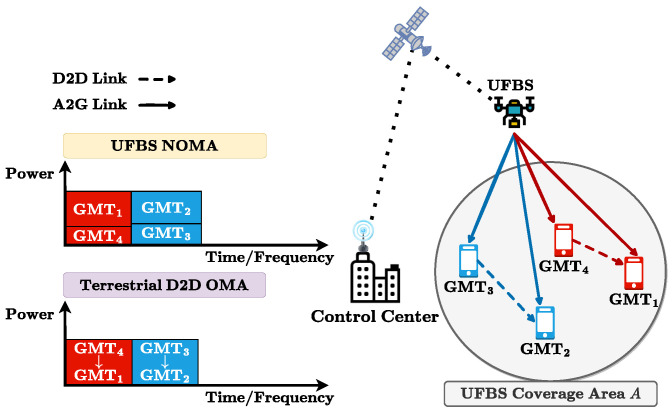
System model.

The wireless network architecture is depicted in Figure 1, where a two-tier heterogeneous network is formed, operating in two different and non-overlapping spectrum bands. From now on, these two ways of communication will be referred to as UFBS NOMA transmission when the GMTs receive the data directly from the UFBS through the NOMA scheme and the D2D cooperative transmission when the GMTs cooperate to improve the overall communication quality. Concerning the UFBS NOMA transmission, all GMTs are served by the UFBS via the air-to-ground (A2G) link, utilizing the NOMA technique according to an optimal power allocation and user pairing strategy [27,28]. More specifically, the total available UFBS’ bandwidth $B_{u}$ is divided into *K* slots, equally distributed to the GMT pairs, as depicted in Figure 1. Each GMT pair *k* (1≤k≤K) consists of a strong ${\mathrm{GMT}}_{i}$ and a weak ${\mathrm{GMT}}_{j}$ ground terminal, with i≠j, which are sharing the same sub-channel in the frequency/time domain. The UFBS classifies the GMTs of each pair as either weak or strong based on the A2G channel conditions. Following the NOMA principle, in each pair of users the strong ${\mathrm{GMT}}_{i}$ first decodes the signal of the weak ${\mathrm{GMT}}_{j}$ from the received superposition-coded signal and then performs successive interference cancellation (SIC) to retrieve its signal. Hence, leveraging this knowledge, the utilization of the D2D cooperative transmission scheme on the ground can further enhance the communication quality of the weak users of the system. Concerning the D2D ground communication procedure, each strong ${\mathrm{GMT}}_{i}$ decodes and forwards (DF) the received UFBS’s signal to the weak ${\mathrm{GMT}}_{j}$ of its pair, thus providing reception diversity through the ground assistance. Consequently, each weak ${\mathrm{GMT}}_{j}$ in each pair will receive two different copies of the same signal, one from the UFBS and the other from its pair, i.e., the strong ${\mathrm{GMT}}_{i}$, which acts as a relay.

From a technical standpoint, the communication system consists of N=2K GMTs, where *K* is the number of GMT pairs and a UFBS located in an *R*-radius circle region of interest *A*. Each ${\mathrm{GMT}}_{l}$ $\left(1\leq l\leq N\right)$ is randomly placed in the region of interest, and its location is expressed as $u_{l}=\left(x_{l}^{u},y_{l}^{u},z_{l}^{u}\right)\in A$. The 3D location of the UFBS is denoted as $p_{1}=\left(x_{1}^{p},y_{1}^{p},z_{1}^{p}\right)\in A$. The UFBS is equipped with an antenna with transmit gain $G_{t}^{u}$, and total available transmit power $P_{u}$. Also, the downlink operating frequency of the UFBS is $F_{u}$. Furthermore, the operating frequency, the total available bandwidth, and the transmit power for the D2D transmission are denoted as $F_{d}$, $B_{d}$, and $P_{d}$, respectively. Moreover, the GMTs are equipped with two antennas, one for the reception of the UFBS’s signals with reception gain $G_{r}^{u}$, and the other for D2D communication, i.e., for transmission and reception, with transmit and receive gain $G_{t}^{d}$=$G_{r}^{d}$. We consider that the common antenna for transmission and reception regarding D2D communication is implemented through a radio frequency (RF) switch. Hence, each GMT can only transmit or receive during a D2D frequency/time slot.

Finally, the seamless communication between the UFBS and the GMTs requires a reliable and efficient backhaul network. In this regard, we propose the use of zero-touch commissioning (ZTC) cloud radio access network (C-RAN) for the UAV backhaul, as it can provide efficient and automated network management [25,29]. The ZTC-C-RAN model comprises a control element that performs the ZTC procedures, including the instantiation, configuration, and synchronization of the UAV and D2D cooperative network as well as the placement of the UFBS in the region of interest *A*. Furthermore, the proposed ZTC-C-RAN is benefited from the satellite communication as a backhaul relay between the UFBS and the control center, providing ultra-reliable low latency communication (URLLC) and enhanced mobile broadband (eMBB) network slices responsible for routing the control and data plane information to the terrestrial and aerial segments of the proposed scheme.

### 2.1. Air-to-Ground and Device-to-Device Channels

The channel between UFBS and its associated GMTs is characterized as an A2G channel. To conduct performance analysis, the channel complex coefficient for each ${\mathrm{GMT}}_{l}$ $\left(1\leq l\leq N\right)$ is denoted as $h_{l}^{u}$, and follows the complex Gaussian distribution with zero mean and unit variance $∼\mathrm{CN}\left(0,1\right)$. Additionally, the path loss attenuation of the UFBS signal is modeled using the elevation angle-based path loss model [25] in an urban environment, and is represented as follows:

(1) $$\begin{array}{c} {\mathrm{PL}}_{l}^{u}\left(h,r_{l}\right)={\mathrm{FSL}}_{l}+\eta_{\mathrm{LoS}}P_{\mathrm{LoS}}\left(h,r_{l}\right)+\eta_{\mathrm{NLoS}}\left(1-P_{\mathrm{LoS}}\left(h,r_{l}\right)\right), \end{array}$$

where ${\mathrm{FSL}}_{l}$ is the free space pathloss given by ${\mathrm{FSL}}_{l}=20\log\left(\frac{4\pi d_{l}F_{u}}{c}\right)$, $d_{l}$ is the transmission distance between UFBS and each ${\mathrm{GMT}}_{l}$ $\left(1\leq l\leq N\right)$, and *c* is the speed of light. In addition, the $\eta_{\mathrm{LoS}}$ and $\eta_{\mathrm{NLoS}}$ coefficients reflect the extra losses for LoS and Non-LoS (NLoS) air-to-ground transmission links, and they depend on the propagation environment. Moreover, $P_{\mathrm{LoS}}$ denotes the probability of the LoS component between the UFBS and each ${\mathrm{GMT}}_{l}$ and is modelled as a function of the altitude *h* of the UFBS and the 2D Euclidean distance $r_{l}$ between the UFBS and each ${\mathrm{GMT}}_{l}$. Hence, $P_{\mathrm{LoS}}$ can be expressed as follows [30]:

(2) $$\begin{array}{c} P_{\mathrm{LoS}}\left(h,r_{l}\right)=\frac{1}{1+a\exp(-b\left(\arctan\left(\frac{h}{r_{l}}\right)-a\right))}, \end{array}$$

where *a*, *b* are parameters determined by the propagation environment. Regarding the D2D link between the strong ${\mathrm{GMT}}_{i}$ and weak ${\mathrm{GMT}}_{j}$ of each pair *k* (1≤k≤K) the multipath fading is modeled by the complex Gaussian distribution with zero mean and unit variance $∼\mathrm{CN}\left(0,1\right)$. The complex channel coefficient for the D2D link is denoted as $h_{k}^{d}$. Moreover the path loss model for the D2D communication of each pair *k* (1≤k≤K), used from [27], is as follows:

(3) $$\begin{array}{c} {\mathrm{PL}}_{k}^{d}\left(d_{k}^{e}\right)=157+\log_{10}\left(d_{k}^{e}\right), \end{array}$$

where $d_{k}^{e}$ is the distance in km between the strong ${\mathrm{GMT}}_{i}$ and the weak ${\mathrm{GMT}}_{j}$ of each pair *k* (1≤k≤K). Furthermore, the A2G and the D2D links under consideration are assumed to be degraded by additive white Gaussian noise (AWGN), which is statistically modeled by the normal distribution $∼N\left(0,\sigma_{q}^{2}\right)$ with q={u,d}. The noise power of the A2G and D2D receivers are given by $N_{u}=k_{B}T_{u}B_{u}$ and $N_{d}=k_{B}T_{d}B_{d}$, respectively; where $k_{B}$ is the Boltzmann constant, and  $T_{u},T_{d}$ are the A2G and D2D receiver system noise temperatures, respectively. Therefore, the corresponding noise variances for each receiver type are $\sigma_{u}=\sqrt{N_{u}}$ and $\sigma_{d}=\sqrt{N_{d}}$.

### 2.2. Transmission and Reception Structure

As previously stated, the UFBS forms *K* user pairs, where each pair *k* $\left(1\leq k\leq K\right)$ consists of one strong ${\mathrm{GMT}}_{i}$ and one weak ${\mathrm{GMT}}_{j}$. Therefore, the wireless communication system under consideration comprises *K* strong GMTs and *K* weak GMTs (2K GMTs in total). Additionally, we assume that the UFBS transmits to the *N* GMTs without any delays. Such an assumption is acceptable for a broadcast system in which the UFBS transmits the information repeatedly, and the GMTs get this information immediately. Thus, the superimposed NOMA signal, transmitted to each pair *k* by the UFBS, is expressed as:

(4) $$\begin{array}{c} x_{k}^{u}=\sqrt{G_{t}^{u}}\left(\sqrt{\alpha_{i}P_{u}}s_{i}+\sqrt{\alpha_{j}P_{u}}s_{j}\right), \end{array}$$

where $s_{i}$, $s_{j}\in C$ are the signals of ${\mathrm{GMT}}_{i}$ and ${\mathrm{GMT}}_{j}$, respectively. Also, $\alpha_{i}$ and $\alpha_{j}$ denote the fraction of the total UFBS transmit power $P_{u}$ allocated to each GMT, with $\alpha_{i}+\alpha_{j}=1$.

The signals received by the strong ${\mathrm{GMT}}_{i}$ and the weak ${\mathrm{GMT}}_{j}$ for each *k* pair are obtained as follows:

(5) $$\begin{array}{c} y_{i}^{u}=\sqrt{\frac{G_{r}^{u}}{{\mathrm{PL}}_{i}^{u}}}h_{i}^{u}x_{k}^{u}+z^{u}, \end{array}$$

(6) $$\begin{array}{c} y_{j}^{u}=\sqrt{\frac{G_{r}^{u}}{{\mathrm{PL}}_{j}^{u}}}h_{j}^{u}x_{k}^{u}+z^{u}, \end{array}$$

where $z^{u}∼N\left(0,\sigma_{u}^{2}\right)$ represents the AWGN of the A2G link. Simultaneously, the received signal at the weak ${\mathrm{GMT}}_{j}$ when the D2D cooperative transmission is activated, is given by the following expression:

(7) $$\begin{array}{c} y_{j}^{d}=\sqrt{\frac{G_{r}^{d}}{{\mathrm{PL}}_{k}^{d}}}h_{k}^{d}x_{j}^{d}+z^{d}, \end{array}$$

where $z^{d}∼N\left(0,\sigma_{d}^{2}\right)$ stands for the AWGN noise in the D2D link. Since we have considered the decode and forward (DF) operation regarding the D2D links, the strong user ${\mathrm{GMT}}_{i}$ of each pair *k* immediately decodes the received UFBS NOMA signal $x_{k}^{u}$ and then estimates the weak user’s signal $\hat{s_{j}}$. Subsequently, the strong user ${\mathrm{GMT}}_{i}$ forwards $\hat{s_{j}}$ to the weak user ${\mathrm{GMT}}_{j}$ through transmitting the signal:

(8) $$\begin{array}{c} x_{j}^{d}=\sqrt{G_{t}^{d}P_{d}}\hat{s_{j}}. \end{array}$$

### 2.3. Signal-to-Interference-Plus-Noise Ratio (SINR) Analysis

In general, for each ${\mathrm{GMT}}_{l}\;\left(1\leq l\leq N\right)$ in the considered communication system, the A2G channel gain is calculated as:

(9) $$\begin{array}{c} \Gamma_{l}^{u}=\frac{G_{t}^{u}G_{r}^{u}}{{\mathrm{PL}}_{l}^{u}N_{u}}{\left|h_{l}^{u}\right|}^{2}, \end{array}$$

including additional gains, losses, and the noise power of the UFBS receiver $N_{u}$. Hence, using (5), the instantaneous signal-to-noise ratio (SNR) $\gamma_{i}^{u}$ of the strong ${\mathrm{GMT}}_{i}$ to detect its own signal $s_{i}$, assuming perfect SIC, is given as follows:

(10) $$\begin{array}{c} \gamma_{i}^{u}=\alpha_{i}P_{u}\Gamma_{i}^{u} \end{array}$$

where $\Gamma_{i}^{u}$ is the A2G channel gain of the strong ${\mathrm{GMT}}_{i}$, which involves the noise power of the UFBS receiver $N_{u}$, as it can be observed in (9). Furthermore, the instantaneous signal-to-interference plus noise ratio (SINR) $\gamma_{k}^{u}$, for detecting the signal $s_{j}$ of the weak user ${\mathrm{GMT}}_{j}$ on the strong user ${\mathrm{GMT}}_{i}$, is expressed as:

(11) $$\begin{array}{c} \gamma_{k}^{u}=\frac{\alpha_{j}P_{u}\Gamma_{i}^{u}}{\alpha_{i}P_{u}\Gamma_{i}^{u}+1}. \end{array}$$

Moreover, the SINR $\gamma_{j}^{u}$ at the weak user ${\mathrm{GMT}}_{j}$, for detecting its own signal $s_{j}$ from the UFBS is obtained by:

(12) $$\begin{array}{c} \gamma_{j}^{u}=\frac{\alpha_{j}P_{u}\Gamma_{j}^{u}}{\alpha_{i}P_{u}\Gamma_{j}^{u}+1}, \end{array}$$

where $\Gamma_{j}^{u}$ is the A2G channel gain for the weak ${\mathrm{GMT}}_{j}$. Furthermore, the SINR $\gamma_{k}^{d}$ at the weak user ${\mathrm{GMT}}_{j}$ for detecting its signal, which is relayed by the strong user ${\mathrm{GMT}}_{i}$ in the same pair *k*, equals:

(13) $$\begin{array}{c} \gamma_{k}^{d}=P_{d}\Phi_{k}, \end{array}$$

where $\Phi_{k}$ is the channel gain of the D2D link between the strong ${\mathrm{GMT}}_{i}$ and the weak ${\mathrm{GMT}}_{j}$ belonging to the same NOMA pair *k* $\left(1\leq k\leq K\right)$ and is expressed as:

(14) $$\begin{array}{c} \Phi_{k}=\frac{G_{t}^{d}G_{r}^{d}}{{\mathrm{PL}}_{k}^{d}N_{d}}{\left|h_{k}^{d}\right|}^{2}, \end{array}$$

### 2.4. Achievable Rates Analysis

As the SINR expressions of the strong ${\mathrm{GMT}}_{i}$ and the weak ${\mathrm{GMT}}_{j}$ for each pair *k* have been determined, it is straightforward to compute the corresponding achievable rates. The theoretical achievable rate of each ${\mathrm{GMT}}_{l}$, when we consider a conventional UAV-OMA transmission scheme, can be mathematically expressed as:

(15) $$R_{l}^{o}=\frac{B_{u}}{2K}\log_{2}\left(1+P_{u}\Gamma_{l}^{u}\right),$$

In contrast, in the case of a UAV-NOMA scheme, the maximum downlink NOMA achievable rates which succeed by the strong ${\mathrm{GMT}}_{i}$ and the weak ${\mathrm{GMT}}_{j}$ through the A2G channel are:

(16) $$R_{i}^{u}=\frac{B_{u}}{K}\log_{2}\left(1+\gamma_{i}^{u}\right),$$

(17) $$R_{j}^{u}=\frac{B_{u}}{K}\log_{2}\left(1+\gamma_{j}^{u}\right),$$

respectively. Moreover, for the strong ${\mathrm{GMT}}_{i}$, the achievable rate of the weak ${\mathrm{GMT}}_{j}$’s signal is equal to:

(18) $$R_{k}^{u}=\frac{B_{u}}{K}\log_{2}\left(1+\gamma_{k}^{u}\right).$$

Also, the maximum achievable rate $R_{k}^{d}$ concerning the established D2D link between the strong user ${\mathrm{GMT}}_{i}$ and the weak user ${\mathrm{GMT}}_{j}$ is expressed as:

(19) $$R_{k}^{d}=\frac{B_{d}}{K}\log_{2}\left(1+\gamma_{k}^{d}\right),$$

Since the weak ${\mathrm{GMT}}_{j}$ can receive its signal directly from the UFBS or via the strong ${\mathrm{GMT}}_{i}$ of the pair it belongs to utilizing the D2D communication capabilities, ${\mathrm{GMT}}_{j}$’s device always chooses to be served by the link that offers the highest achievable rate. Thus, it holds that the maximum achievable rate of each weak ${\mathrm{GMT}}_{j}$ that belongs to the NOMA pair *k*, combining the UAV-NOMA with cooperative D2D scheme, can be calculated as follows:

(20) $$R_{j}^{\mathrm{COPD}2D}=\max\left(R_{j}^{u},\Lambda_{j}\right),$$

where $\Lambda_{j}$ is the achievable rate through the D2D communication with the strong ${\mathrm{GMT}}_{i}$. In fact, the weak ${\mathrm{GMT}}_{j}$’s signal is decoded on the strong ${\mathrm{GMT}}_{i}$, and the D2D communication provides the channel to forward this decoded signal from the strong ${\mathrm{GMT}}_{i}$ to the weak ${\mathrm{GMT}}_{j}$. As a result, the weak ${\mathrm{GMT}}_{j}$ can never receive a rate greater than $R_{k}^{u}$, meaning that $\Lambda_{j}\leq R_{k}^{u}$. Essentially, the quality of the D2D communication will determine whether the weak ${\mathrm{GMT}}_{j}$ will enjoy the maximum possible rate $R_{k}^{u}$ or less. Specifically, we can recognize the following cases:

**Case** **1.** *The D2D channel is profitable for the weak user, i.e., $R_{k}^{d}\geq R_{k}^{u}$, and the achievable rate of the weak user is $\Lambda_{j}=R_{k}^{u}$. This happens because the weak user can never receive a rate greater than the achievable decoding rate of its signal on the strong user.*

**Case** **2.** *The D2D channel is not profitable for the weak user, i.e., $R_{k}^{d}<R_{k}^{u}$, and the achievable rate of the weak user is equal to the transmission rate that the D2D communication can provide, i.e., $\Lambda_{j}=R_{k}^{d}$. In this case, we observe that the achievable rate of the weak user is limited based on the capabilities of the D2D communication channel.*

Based on the above cases concerning the use of D2D communication for receiving the signal on the weak user, we observe that the minimum rate between the achievable rates $R_{k}^{d}$ and $R_{k}^{u}$ is always selected. Therefore, in the case where D2D communication is used, it follows that the achievable rate of the weak user is equal to $\Lambda_{j}=\min\left(R_{k}^{u},R_{k}^{d}\right)$. By substituting $\Lambda_{j}$ in (20):

(21) $$R_{j}^{\mathrm{COPD}2D}=\max\left(R_{j}^{u},\min\left(R_{k}^{u},R_{k}^{d}\right)\right).$$

Utilizing the UAV-NOMA and D2D-aided scheme the total sum rate which is succeeded on each pair *k* is equal to:

(22) $$R_{k}=R_{i}^{u}+R_{j}^{\mathrm{COPD}2D}.$$

Therefore, the total system sum rate that can be achieved by utilizing the aforementioned cooperative scheme is:

(23) $$R_{s}=\sum_{k=1}^{K}R_{k}$$

### 2.5. User Pairing Policy

So far, we have noted that the system’s GMTs are separated into *K* groups of two members each, but we have not specified how the GMTs are allocated to each group. Hence, in this sub section, we propose the maximum weight perfect matching (MWPM) pairing policy which takes into account both the A2G and D2D channel conditions. The primary objective is to maximize the system’s total sum rate. Therefore, a matching technique must be implemented between the GMTs in order to discover those user pairs that optimize the system’s overall sum rate. The MWPM method generates N2 pairings between the *N* GMTs and retains the *K* that maximize the system sum rate. For this purpose, it is necessary to define a binary matrix Θ that represents the pairing relationship between the GMTs as follows:

(24) $$\theta_{i,j}=\left\{\begin{array}{ccccc} \; & 1 & {\mathrm{GMT}}_{i} & \mathrm{paired}\;\mathrm{with} & {\mathrm{GMT}}_{j}\\ \; & 0 & \mathrm{otherwise} \end{array}\right.$$

The dimension of the pairing matrix Θ that is retrieved from the MWPM method is equal to N×N. Moreover the diagonal elements of the pairing matrix Θ are all equal to zero because one GMT cannot pair itself. Also, due to the fact that the matrix components $\theta_{i,j}$ and $\theta_{j,i}$ both pertain the same GMT pairing, it can also be argued that $\theta_{i,j}=\theta_{j,i}$. Therefore, the MWPM pairing policy can be expressed as the following maximization problem:

(25) $$\begin{array}{cc} \max_{\theta_{i,j}}\; & \sum_{i=1}^{N}\sum_{j=i+1}^{N}\theta_{i,j}\left(R_{i}^{u}+R_{j}^{\mathrm{COPD}2D}\right),\\ s.t.\; & \sum_{j=1}^{i-1}\theta_{j,i}+\sum_{j=i+1}^{N}\theta_{i,j}=1,\forall i=1,2,3,\cdots ,N\\ \; & \theta_{i,j}\in \left\{0,1\right\},\;1\leq i,j\leq N. \end{array}$$

The maximization problem (25) can be regarded as a matching problem in a fully connected undirected graph $G\left(V,E\right)$, where the total number of vertices is equal to the total number of GMTs $\left|V\right|=N$. E is the set of all feasible edges $\theta_{i,j}$, connecting all users to each other with i≠j and $i,j=\left\{1,2,...,N\right\}$. In order to solve this issue optimally, we use the Blossom algorithm to obtain an optimal pairing strategy between the GMTs [31].

### 2.6. Power Allocation Strategy

Concerning the UFBS NOMA transmission, the objective is to maximize the sum rate of each pair of GMTs under the condition that both GMTs enjoy at least the rate utilizing the conventional UFBS OMA transmission. This is an optimization problem which is mathematically expressed as follows:

(26) $$\begin{array}{cc} \max_{\alpha_{i}}\; & R_{i}^{u}+R_{j}^{u},\\ s.t.\; & R_{i}^{u}\geq R_{i}^{o},\\ \; & R_{j}^{u}\geq R_{j}^{o},\\ \; & 0\leq \alpha_{i}\leq 1. \end{array}$$

The solution to this problem has been obtained in [26,27] by identifying the optimal value of $\alpha_{i}$, as:

(27) $$\alpha_{i}=\frac{\sqrt{1+\Gamma_{j}^{u}P_{u}}-1}{\Gamma_{j}^{u}P_{u}}.$$

To conclude, in Table 1, the definitions of most of the parameters involved in this study are included.

## 3. UFBS Placement Procedure

In this section, we analyze the placement procedure of the UFBS in the region of interest *A*. For this purpose, we propose an UFBS placement procedure that is divided into two sub-processes. The first sub-process aims to find the 2D plane position of the UFBS. For this purpose, k-means and k-medoids algorithms are exploited and assessed [7,9]. The second sub-process seeks to discover the UFBS’s height aiming to improve coverage and communication quality, thus determining its location in the three-dimensional space.

### 3.1. 2D UFBS Placement

#### 3.1.1. k-Means Analysis and Setup

This sub-subsection describes the UFBS 2D placement procedure utilizing the k-means algorithm. In more detail, the k-means algorithm is fed with the coordinates $u_{l}$$\left(1\leq l\leq N\right)$ of all GMTs located within the region of interest *A*. Subsequently, the algorithm groups the users into a cluster and returns as output the centroid point $p_{1}^{c}\in A$ where $p_{1}^{c}=\left(x_{1}^{p_{1}^{c}},y_{1}^{p_{1}^{c}},z_{1}^{p_{1}^{c}}\right)$. The goal of the k-means method is to minimize the centroid-point to group distances metric, expressed as $\sum_{u_{l}\in U}{∥u_{l}-p_{1}^{c}∥}^{2}$. In particular, this expression represents the objective function of the following minimization problem:

(28) $$\begin{array}{c} \underset{p_{1}^{c}\in A}{\arg\min}\sum_{u_{l}\in U}{∥u_{l}-p_{1}^{c}∥}^{2}. \end{array}$$

Therefore, the UFBS should be placed in $p_{1}^{c}$ to achieve improved communication quality. The operation of the 2D UFBS placement process using the k-means algorithm is summarized in Algorithm 1.
**Algorithm 1** 2D UFBS placement process through the k-means algorithm1:**input**: The set of coordinates of all GMTs $U=\{u_{1},u_{2},\cdots ,u_{N}\}$, and the number of UFBSs Y2:$\epsilon =10^{-6}$3:*t* = 04:Initialize Y centroid points $C^{t}=\left\{p_{1}^{c},p_{2}^{c},...,p_{Y}^{c}\right\}\subseteq U$, randomly5:**repeat**6:    $S_{k}$ = $\emptyset ,\forall_{k}=1,2,\cdots ,Y$7:    **for** i←1 to *N* **do**8:        $k^{*}=\underset{k=1\cdots Y}{\arg\min}∥u_{i}-C_{k}^{t}∥$9:        $S_{k^{*}}=S_{k^{*}}\cup \left\{u_{i}\right\}$10:    **end for**11:    **for** k←1 to Y **do**12:        $C_{k}^{t}=\frac{1}{|S_{k}|}\sum_{u_{l}\in S_{k}}u_{l}$.13:    **end for**14:    *t* = *t* +115:**until** $C^{t}-C^{t-1}\leq \epsilon$16:**output**: A set of centroid points that the Y UFBSs will be deployed $C^{t}$.

For simplicity, it is assumed that the number of UFBS Y=1. However, as can be shown in Algorithm 1, the k-means algorithm can be straightforwardly applied to scenarios with Y>1. Hence, in our case, the centroid $p_{1}^{c}$ is given by the following three steps:

**Step** **1:** Determine the coordinate $Y_{u}$ of the UFBS as follows: $Y_{u}=\frac{\sum_{i=1}^{N}y_{i}^{u}}{N}$
**Step** **2:** Determine the coordinate $X_{u}$ of the UFBS as follows: $X_{u}=\frac{\sum_{i=1}^{N}x_{i}^{u}}{N}$
**Step** **3:** Configure the point $p_{1}^{c}$ that the UFBS should be placed as follows: $p_{1}^{c}=\left(X_{u},Y_{u},h\right)$, where *h* is the initial height of the UFBS before the 3D UFBS placement procedure.

Finally, it is essential to acknowledge that the choice of the optimal number of clusters for a clustering problem is not straightforward and may be influenced by a range of factors, including the specific requirements and objectives of the analysis, as well as the inherent properties of the data. Within the context of our system model, the user locations are randomly distributed within a circular region of interest, forming a single cluster. This characteristic of the data renders the choice of Y equal to 1 in k-means clustering a sensible and appropriate decision, as it adequately captures the underlying structure of the data. The resulting cluster is representative of the overall distribution of users and adequately reflects the inherent properties of the dataset. In this particular scenario, using a single cluster is sufficient to accurately and effectively represent the nature of the user distribution and therefore is a suitable approach to analyze the data [32].

#### 3.1.2. k-Medoids Analysis and Setup

In this sub-subsection, the basic principles of the k-medoids algorithm are presented. The k-medoids method can be used for the 2D placement of the UFBS in *A* in the same fashion as k-means. However, the way that the UFBS placement point $p_{1}$ is selected differs between the two approaches. As previously stated, in the k-means UFBS placement scheme, the centroid point $p_{1}^{c}$ is the empirical mean of the coordinates *U* of the GMTs in *A*. However, in k-medoids, it is one of the actual ${\mathrm{GMT}}_{l}$ $\left(1\leq l\leq N\right)$, and it is called medoid point $p_{1}^{m}$. Specifically, in k-means, the point-to-group-centroid distance is assessed concerning a virtual point $p_{1}^{c}\in A$, while in k-medoids, it is measured concerning one of the actual data points $u_{l}\in A$$\left(p_{1}^{m}=u_{l}\right)$ where $\left(1\leq l\leq N\right)$, i.e., actual GMTs location. Similarly to the k-means algorithm, the goal of the k-medoid method is to minimize the medoid-point to group distances metric, expressed as $\sum_{u_{l}\in U}{∥u_{l}-p_{1}^{m}∥}^{2}$ by solving the following minimization problem:

(29) $$\begin{array}{c} \underset{p_{1}^{m}\in A}{\arg\min}\sum_{u_{l}\in U}{∥u_{l}-p_{1}^{m}∥}^{2}. \end{array}$$

The operation of the 2D UFBS placement process using the k-medoids algorithm is summarized in Algorithm 2.

In the same manner with k-means, it is assumed that the number of UFBS Y = 1. However, as can be shown in Algorithm 2, the k-medoids algorithm can be straightforwardly applied to scenarios with Y>1. Additionally, Algorithm 3 is the modified version of Algorithm 2 for the special case where Y=1.
**Algorithm 2** 2D UFBS placement process through the k-medoids algorithm1:**input**: The set of coordinates of all GMTs $U=\{u_{1},u_{2},\cdots ,u_{N}\}$, and the number of UFBSs Y2:$\epsilon =10^{-6}$3:*t* = 04:Initialize Y medoid points $C^{t}=\left\{p_{1}^{m},p_{2}^{m},...,p_{Y}^{m}\right\}\subseteq U$, randomly5:$S_{k}$ = $\emptyset ,\forall_{k}=1,2,\cdots ,Y$.6:**for**i←1 to *N* **do**7:    $k^{*}=\underset{k=1\cdots Y}{\arg\min}∥u_{i}-C_{k}^{t}∥$8:    $S_{k^{*}}=S_{k^{*}}\cup \left\{u_{i}\right\}$9:**end for**10:$A^{t}=\sum_{k=1}^{Y}\sum_{u_{l}\in S_{k}}{∥u_{i}-C_{k}^{t}∥}^{2}$11:**repeat**12:    **for** k←1 to Y **do**13:        **for** i←1 to *N* **do**14:           **if** $u_{i}\notin C^{t}$ **then**15:               Swap the role of $C_{k}^{t}$ with $u_{i}$16:               Repeat steps 6 to 917:               $B=\sum_{k=1}^{Y}\sum_{u_{l}\in S_{k}}{∥u_{i}-C_{k}^{t}∥}^{2}$18:               **if** $B<A^{\left[t\right]}$ **then**19:                   *t* = *t* +120:                   $C_{k}^{t}$ = $u_{i}$21:                   $A^{t}=B$22:               **end if**23:           **end if**24:        **end for**25:    **end for**26:**until** $C^{t}-C^{t-1}\leq \epsilon$27:**output**: A set of centroid points that the Y UFBSs will be deployed $C^{t}$.

**Algorithm 3** 2D UFBS placement process through the k-medoids algorithm with Y = 1
1:**input**: The set of coordinates of all GMTs $U=\{u_{1},u_{2},\cdots ,u_{N}\}$2:$B_{k}$ = $0,\forall_{k}=1,2,\cdots ,N$3:**for**i←1 to *N* **do**4:    $B_{i}=\sum_{j=1}^{N}{∥u_{i}-u_{j}∥}^{2}$5:
**end for**
6:

$j^{*}=\underset{i=1\cdots N}{\arg\min B_{i}}$

7:**output**: The medoid point that the UFBS will be deployed $u_{j^{*}}$.


### 3.2. 3D UFBS Placement

Following the determination of the UFBS’s 2D deployment location, the 3D UFBS placement procedure adjusts the UFBS’s altitude to provide the highest quality of service to GMTs within the area of interest *A*. Thus, the farthest ${\mathrm{GMT}}_{l}$ from the point $p_{1}$ where the UFBS is finally placed should be identified, according to the horizontal two-dimensional distance $r_{l}$. After that, the convenient height for the critical point $p_{1}$ is found by solving the following equation using (1):

(30) $$\begin{array}{c} \frac{\partial {\mathrm{PL}}_{l}^{u}\left(h,r_{l}^{p_{1}}\right)}{\partial h}=0. \end{array}$$

For the considered A2G path-loss model, as the altitude of the UFBS increases the path loss initially decreases and then increases again. This behavior can be attributed to the dependence of the particular A2G model on the elevation angle and the distance between the UFBS and each ${\mathrm{GMT}}_{l}$. As the height of the UFBS increases the elevation angle also increases, leading to an increased probability of line-of-sight, i.e., obscurance by buildings and other surrounding objects is reduced. Based on this behavior, the A2G path loss ${\mathrm{PL}}_{l}^{u}$ function is convex [25]. Thus, it can be deduced that the global minimum is consistently located at the critical point which can be derived through the Equation (30).

### 3.3. Computational Complexity of k-Means and k-Medoids Algorithms

Another crucial aspect is to estimate the computational complexity of the examined k-means and k-medoids algorithms based on their respective methods as described in Algorithms 1 and 2, respectively. K-means is a centroid-based algorithm, and k-medoids is a medoid-based algorithm.

The computational complexity of the k-means algorithm has been proven to be O(nkId), where n is the number of data points, k is the number of clusters, I is the number of iterations, and d is the number of dimensions [33]. It uses the mean of the data points to calculate the cluster centroid and updates the assignment of the data points to the closest cluster centroid. The algorithm requires multiple iterations until convergence. The time complexity of the k-means algorithm is affected by the number of data points, the number of clusters and the number of dimensions.

The computational complexity of the k-medoids algorithm has been proven to be $O(k{\left(n-k\right)}^{2}I)$, where n is the number of data points, k is the number of clusters, and I is the number of iterations [34]. K-medoids selects a single data point as the representative of a cluster, known as the medoid, and updates the assignment of the data points to the closest medoid. The algorithm requires multiple iterations until convergence. The time complexity of the k-medoids algorithm is affected by the number of data points, clusters, iterations, and the distance metric used.

In summary, both algorithms have a polynomial time complexity, and the main difference is that k-means use centroids, and k-medoids use medoids as the center of the cluster. As a result, the k-means is sensitive to the initial choice of centroids, while k-medoids is less sensitive and tends to find the global optimum more quickly.

## 4. ANN-Based Placement Scheme Selection

The main difference between the two algorithms mentioned before, is that the virtual centroid point $p_{1}^{c}\in A$ given from the k-means where the UFBS will be placed, will be equidistant from all GMTs. Conversely, the medoid point $p_{1}^{m}\in A$ given from the k-medoids will be a GMT location within the region of interest that will minimize the objective function (see (29)). Consequently, if the GMTs are spread equally in the area of interest, the $p_{1}^{c}$ point provided by k means will improve the channel quality of GMTs, since the distances of the GMTs from the UFBS will be almost identical and the LoS probability will be significantly high. On the contrary, if a GMT is remote (outlier), the k-means algorithm will try to find the point $p_{1}^{c}$ equidistant from every GMT, detaching it quite a bit from the majority of GMTs and thus increasing the GMTs’ propagation losses. In contrast, the k-medoids through the proposed $p_{1}^{m}$ point reduce the point-to-group-centroid distances, achieving higher A2G channel gains and increasing the QoS of the overall system.

**Figure 2:**
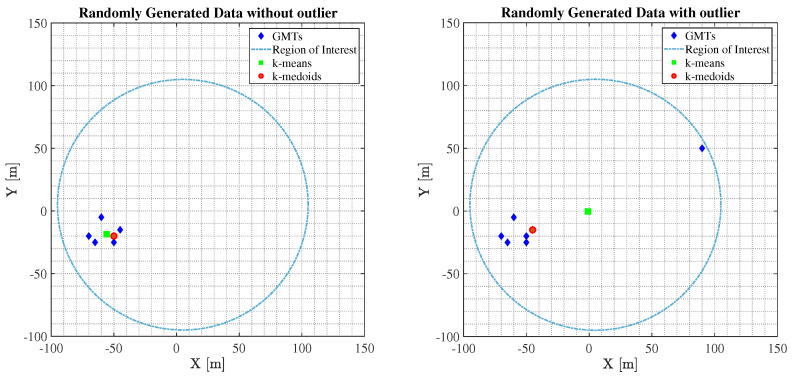
Comparisons between the k-means and k-medoids regarding the UFBS placement procedure.

To better highlight the advantages of each algorithm, let us consider a toy network with GMTs located in the 2D plane as depicted in Figure 2. Focusing on Figure 2 on the right, the group of GMTs in the right form a cluster, while the rightmost GMT is an outlier. The $p_{1}^{c}\in A$ point proposed by the k-means is greatly influenced by the outlier and thus cannot represent the correct cluster center. In contrast, the medoid point $p_{1}^{m}\in A$ provided by k-medoids is robust to the outlier and correctly represents the cluster center. On the contrary, regarding Figure 2 on the left, we notice that there is no remote GMT, and everyone is close to each other, forming a cluster of GMTs. Consequently, the $p_{1}^{c}\in A$ proposed from the k-means is equidistant from all GMTs, thus increasing the channel gain compared to the $p_{1}^{m}\in A$, which is not equidistant from all GMTs offered from the k-medoids algorithm.

Motivated by this observation, the PSS can be regarded as a supervised classification problem, where it can be approximated through the utilization of a fully connected artificial neural network (ANN) to enhance the overall system QoS. Since an ANN model learns how to efficiently match predictions to patterns seen during the training method, a data set containing various features that affect the A2G transmission should be created. To this end, this section presents the data set generation procedure, the date prepossessing, and the hyper-parameter tuning of the ANN model.

### 4.1. Data Set Generation

In this subsection, the dataset generation procedure concerning the training of the ANN model is presented. The objective of the ANN model is to predict the UFBS placement method to enhance the overall communication quality according to specific key performance indicator (KPI). In this work, the considered KPI that should be improved is the total system sum rate, $R_{s}$, given in expression (23). Hence, the optimization problem that the ANN model aims to solve is represented by Equation (23), which expresses the objective function that the ANN model seeks to maximize. This can be achieved through the ability of a well trained ANN model to recognize patterns, indicating when each method should be conducted to achieve the highest system sum rate. Using Equation (23) as a KPI for dataset generation ensures that the generated data is relevant and valuable for training and evaluating ANN models. Furthermore, incorporating a KPI directly aligned with the problem being addressed can guarantee that the model is configured optimally for the targeted classification task and exhibit superior performance for the specific issue [35]. Hence, considering the k-means and the k-medoids algorithms, the ANN should determine which of these two UFBS placement methods will achieve the highest $R_{s}$. Furthermore, the calculation of $R_{s}$ involves various transmission parameters of the considered wireless communication system presented in Section 2, such as the 3D location of the UFBS, as well as the A2G propagation model. Therefore, all these aspects should be carefully considered during the training procedure of the ANN model.

In general, optimizing the total system sum rate, i.e., the $R_{s}$, can offer valuable insights into the optimal allocation of system resources, including bandwidth and transmit power [36]. In the context of a UAV-NOMA and D2D cooperative network, optimizing $R_{s}$ can help identify the most effective resource allocation strategies for achieving optimal system performance. For instance, optimizing the total system sum rate allows the cooperative scheme to allocate bandwidth and power to UAVs and D2D users to maximize the total data rate transmitted over a given period. In addition, this optimization process can consider the physical layer parameters of the UAV and D2D users, including their communication requirements. For example, UAVs may require higher power allocations to maintain stable connections due to their altitude. Additionally, the distance of D2D users from the UFBS can impact their channel conditions and overall communication performance. Considering these physical layer parameters during the optimization process, the system can allocate resources more efficiently and effectively, leading to improved overall performance. In summary, optimizing the sum rate in a UAV-NOMA and D2D cooperative network can help achieve the best use of resources and enhance the system’s overall performance. It is noted that the proposed capacity based optimization of the $R_{s}$ can be considered as the upper bound on the maximum amount of data that can be reliably transmitted over a communication channel as the size of the channel goes to infinity. However, achieving this limit is often difficult in real world scenarios due to practical constraints such as noise and interference in the channel.

Focusing on the data set generation process, Monte Carlo simulations were carried out using Matlab^©^ (MATLAB (Version R2021a) [Computer software]. MathWorks, Natick, MA, USA) software to conduct the entire training data set *D*, following the system model described in Section 2 and depicted in Figure 1. More specifically, in each simulated transmission frame, the GMTs are generated randomly following the uniform distribution into the circular region of interest, while the UFBS is placed through the two unsupervised algorithms mentioned above. It is noted that all GMTs are served by the UFBS via the A2G link, utilizing the NOMA technique, while the D2D cooperative transmission is activated to improve the overall communication quality. The A2G and D2D channel gains are generated based on expressions (9) and (14), respectively, while the urban environment parameters are given in Section 5. Concerning the dataset format, it can be expressed as $D=\{\left(x_{i},y_{i}\right)\}$ with i=1,⋯,d, where *d* is the total number of instances. Also, $x_{i}\in R^{w}$ is the input vector of the *i*-th instance comprised of *w* features and $y_{i}\in \left\{k-\mathrm{means},\;k-\mathrm{medoids}\right\}$ is the class of $x_{i}$. In the following, the input features vector $x_{i}$ consists of eight parameters, i.e., w=8, that affect the placement procedure of the UFBS and are presented in detail in Table 2. Moreover, for the computation of class $y_{i}$, we evaluate the total system sum rate $R_{s}$ in each simulated frame (see Equation (23)) for each UFBS placement procedure. Thus, the class value of the *i*-th instance, $y_{i}$, is determined as the placement method that achieved the highest $R_{s}$.

To precisely train the ANN model and to prevent over-fitting and under-fitting issues, the entire data set *D* is divided into training, validation, and testing subsets using the data splitting approach. A popular strategy for data partitioning is to use 70–80% of the entire dataset for training, with the remaining proportion used to improve and assess the trained models. Consequently, 70% of the total samples are chosen for the training phase, 15% for validation, and the remaining 15% for testing the proposed ANN model [37]. The training set is used to train the ANN, the validation set is used to evaluate the performance of the ANN during training, and the testing set is used to evaluate the performance of the ANN after training.

### 4.2. Data Pre-Processing

The effectiveness of an ANN is highly dependent on the quantity and quality of training data. Consequently, regardless of which classifier is used, inferior models are generated if the training data are inaccurate. In light of the above assertion, stratified sampling and data normalization procedures are utilized to obtain the most incredible performance of the ANN model.

**Figure 3:**
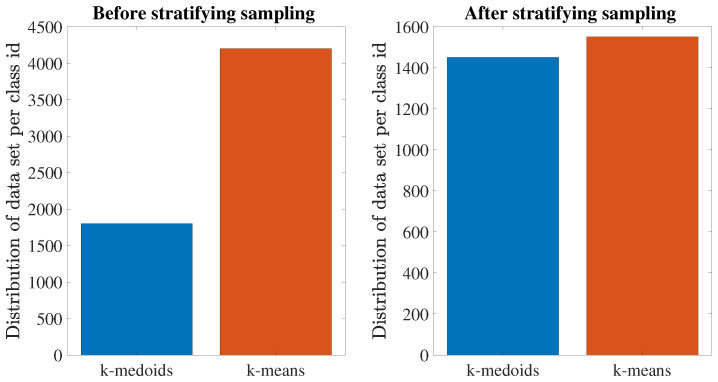
Distribution of data set per class.

As an essential data pre-processing step, instance selection is employed not only to cope with the infeasibility of learning from massive data sets, but also to reduce the risk of the ANN model tending towards the majority and avoid coming up with what is known as the accuracy paradox [38]. For this purpose, stratifying sampling is applied. Hence, the overall training set is reduced, and the class values are uniformly distributed in the training sets, as shown in Figure 3. After removing redundant instances per class values, 3000 data samples were collected, which means a 50% reduction of the initial 6000 raw data samples. In addition, an ANN model cannot attain optimal performance if the feature values are in different units and scales.

In order to resolve these challenges, it is necessary to use a normalizing technique that eliminates the effects of those mismatches. Using this approach, the values of the dataset’s features are scaled into a given range while keeping the original dataset’s overall distribution and ratios. Hence, before the training phase, all input features were normalized for this purpose. The formula for normalizing is as follows:

(31) $$X_{\mathrm{norm}}=\frac{X-X_{\min}}{X_{\max}-X_{\min}}$$

where *X* is a value of the corresponding feature under normalization, $X_{\max}$ and $X_{\min}$ are the maximum and the minimum value of this feature, respectively, and $X_{\mathrm{norm}}\in \left[0,1\right]$ is the final normalized value [37].

### 4.3. ANN Model Construction

ANN has the most hyper-parameters to be tuned among all the ML algorithms. Consequently, this subsection provides a concise but adequate description of the standard hyperparameters of an ANN model and their tuning.

The first step in hyperparameter tuning is finding the layer type [39]. Since non-linear data collection is used in this study, we investigate a fully connected multi-layer perceptron (MLP) network in which the input from the dataset propagates in one direction through one or more hidden layers. Therefore, using the normalized feature vectors obtained through (31) and their corresponding labels, we can build an ANN model consisting of one input layer, $l_{i}=1$, $l_{h}\in \left\{1,2,...,L\right\}$ hidden layers, and one output layer $l_{o}=1$ for the PSS prediction. The $l_{i}$ layer consists of $m_{i}=8$ neurons which represent the input features vector $x_{i}$ for the ANN:

(32) $$x_{i}=\left[D_{\mathrm{ptc}},D_{\mathrm{ptm}},r_{\min}^{p_{c}},r_{\max}^{p_{c}},r_{\min}^{p_{m}},r_{\max}^{p_{m}},{\mathrm{PL}}_{\mathrm{sum}}^{p_{c}},{\mathrm{PL}}_{\mathrm{sum}}^{p_{m}}\right].$$

Each term mentioned in (32) is a real number and is described in detail in Table 2. Moreover, the $l_{o}$ consists of $m_{o}=2$ neurons, which is the total number of classes that we want to predict. The number of neurons $m_{h}$ per hidden layer can be determined as [40]:

(33) $$m_{h}=\left[\frac{m_{i}+\sqrt{d}}{l_{h}}\right]$$

Consequently, if there is one hidden layer ($l_{h}=1$), the number of neurons is 63 according to (33). Similarly, the number of neurons for two hidden layers ($l_{h}=2$) is 31.5 per layer, resulting in the selection of 32 and 31 neurons for the first and second hidden layers. Additionally, we model ANN with $l_{h}=3$ and $l_{h}=4$ hidden layers, and the number of neurons per each hidden layer is listed in Table 3.

The following step in hyperparameter tuning concerning ANN models is to determine the activation and the loss function. In this study, the rectified linear unit (ReLU) activation function is employed in hidden layers. It is easy to build and overcome the constraints of widely used activation functions like Sigmoid and Tanh. Furthermore, since PSS may be seen as a binary classification problem, the output layer activation function is SoftMax. Regarding the loss function, cross-entropy is utilized since it is the most widely used for classification problems. Therefore, in order to find the best ANN hyperparameters, the selected loss function should be minimized. The minimization of the loss function is achieved through gradient descent (GD) with momentum backpropagation. The momentum term navigates the GD along the relevant direction and softens the oscillations in irrelevant directions. For this purpose, the grid search method is utilized. Accordingly, the momentum is tested for values between 0.2 and 1 with a step of 0.1. In the last phase of hyperparameter tuning, the learning rate and the number of epochs are chosen. The learning rate is evaluated for values between 0.001 and 0.1 with a step of 0.001, while the number of epochs range is set to be from 1 to 1000. In addition, the early stopping criterion is used to improve the model’s generalization capability and minimize overfitting. Finally, in Table 4, all the finalized hyperparameters are listed for ANNs methods derived throughout the training, validation, and testing process.

**Figure 4:**
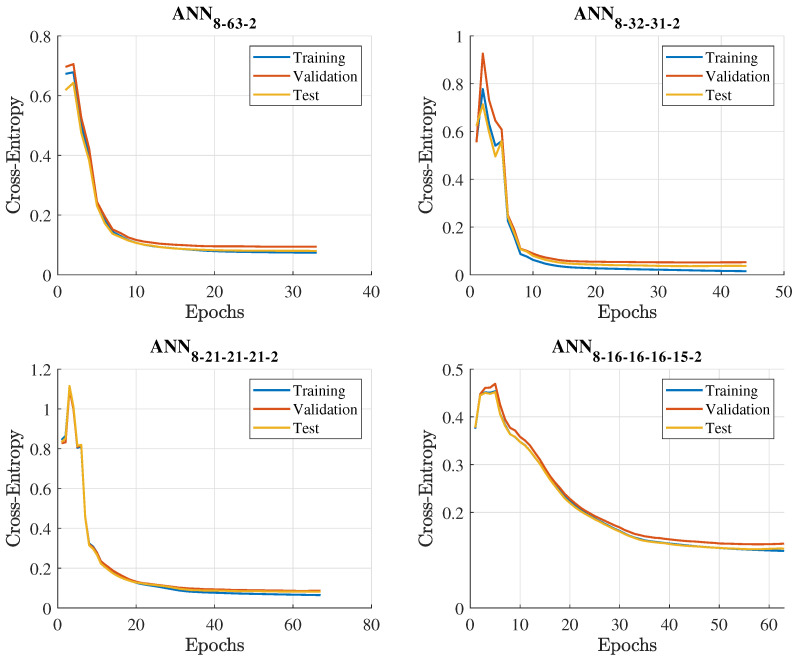
Loss convergence progression versus epochs for the training, validation, and testing phase of all the introduced ANNs.

Figure 4 presents the evaluation of the training, validation, and testing phases in terms of the loss function versus the number of epochs. In essence, the number of epochs directly affects the adopted method’s convergence. The low number of epochs entails that the algorithm may converge at a local minimum. Nonetheless, too many epochs may lead to over-learning. The results in Figure 4 concerning the modelled ANNs prove that the loss function for all processes, i.e., training, validation, and testing, converges smoothly, obtaining constant loss values and reaching the global minimum in a short period. The acquired global minimum loss for the convergence during the testing phase, and the corresponding epoch values are listed in Table 3. According to Table 3, ANN with two hidden layers demonstrates the best performance among all the examined ANN techniques, providing the minimum loss score of 0.06. Furthermore, for each ANN layout, the training time is also recorded. Specifically, the training times for ANN with one, two, three, and four hidden layers are 0.81, 0.92, 1.4, and 1.6 seconds, respectively. Comparing the training time of the assessed ANNs models, it is evident that the training time depends directly on the applied layout structure. Finally, the conventional time complexity (TTC) for any ANN layout is $O(n^{3})$ [37]. The TTC represents the standard theoretical asymptotic complexity, which takes into account only the training samples n. It only examines training samples, since the training phase is the most time-consuming operation in ML algorithms and occurs offline, and not in real-time scenarios.

### 4.4. ANN Model Selection

This section presents the evaluation results obtained from the ANNs methods for the testing set. The evaluation of the ANNs methods and, by extension, the choice of the ANN algorithm to solve the PSS classification problem is achieved based on the accuracy, precision, recall, and F1 score performance metrics.

Specifically, accuracy, precision, recall, and F1 score are commonly used evaluation metrics for assessing the performance of ML models, particularly in classification tasks. These metrics are calculated based on the number of true positive (TP), true negative (TN), false positive (FP), and false negative (FN) predictions made by the model. Accuracy is the proportion of correct predictions made by the model out of all predictions made. In the context of sum-rate maximization, a high accuracy score would indicate that the ANN can predict the best PSS more often accurately, and it is calculated as follows:

(34) $$\mathrm{Accuracy}=\frac{\mathrm{TP}+\mathrm{TN}}{\mathrm{TP}+\mathrm{TN}+\mathrm{FP}+\mathrm{FN}}$$

Precision is the proportion of true positive predictions made by the model out of all positive predictions made. For example, in the context of sum-rate maximization, a high precision score would indicate that when the ANN predicts a PSS, it is more likely to be the best prediction that maximizes the system sum rate, and it can be expressed as follows:

(35) $$\mathrm{Precision}=\frac{\mathrm{TP}}{\mathrm{TP}+\mathrm{FP}}$$

Recall (also known as sensitivity or true positive rate) is the proportion of true positive predictions made by the model out of all actual positive cases. In term of sum-rate maximization, a high recall score would indicate that the ANN is able to find more of the actual PSS solutions, and it is calculated as follows:

(36) $$\mathrm{Recall}=\frac{\mathrm{TP}}{\mathrm{TP}+\mathrm{FN}}$$

F1 score is a harmonic mean of precision and recall. In the context of sum-rate maximization, a high F1 score would indicate that the ANN has a good balance of precision and recall, making fewer false PSS predictions while also identifying most of the relevant cases. It is calculated as:

(37) $$F1=2\times \frac{\mathrm{Precision}\times \mathrm{Recall}}{\mathrm{Precision}+\mathrm{Recall}}$$

**Figure 5:**
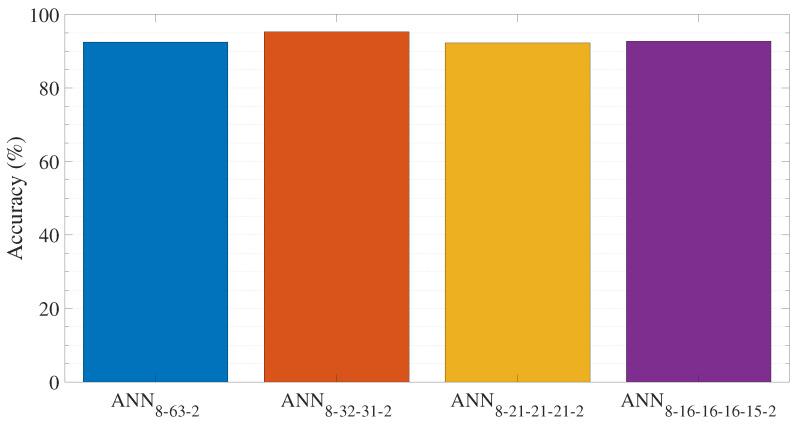
Accuracy comparison between the different ANN layouts.

**Figure 6:**
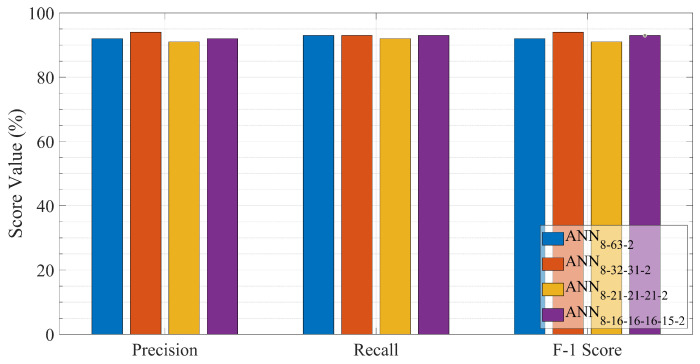
F1-score, precision, and recall performance measurements of all ANN layouts.

Figure 5 and Figure 6 present the evaluation results obtained from the ANN methods for the testing set. Accuracy, precision, recall, and the F1 score are used to evaluate the ANN’s approaches. More specifically, the accuracy of each ANN model is depicted in Figure 5, while Figure 6 illustrates the mean precision, recall, and F1-score obtained from each ANN method. The classification accuracy in Figure 5 reveals that the best prediction is achieved through the ANN with two hidden layers ${\mathrm{ANN}}_{8-32-31-2}$. Comparing the performance of the different ANN layouts, the prediction accuracy decreases until the neural network reaches two hidden layers in depth. Then, by extending the depth of the ANNs to more than two hidden layers, the accuracy is diminished. Specifically, the prediction accuracy increases from 92.5% for a single hidden layer (${\mathrm{ANN}}_{8-63-2}$) to 95.32% for a two-layered (${\mathrm{ANN}}_{8-32-31-2}$) and then decreases to 92.3% and 92.7% for a three (${\mathrm{ANN}}_{8-21-21-21-2}$) and four-layered (${\mathrm{ANN}}_{8-16-16-16-15-2}$) structure, respectively. As can be observed in Figure 6, the assessed ANN models exhibit exceptional performance with an F1-score greater than 91%, maintaining an average accuracy and average recall greater than 91%. Among the evaluated ANNs, the neural network with two hidden layers ${\mathrm{ANN}}_{8-32-31-2}$ achieves the best prediction result. The specific model yields a mean precision of 94.12%, a mean recall of 93.14%, and an average F1-score of 93.63%. Hence this level of accuracy in a balanced data set implies that the model has recognized and formed strong correlations between features and class and has avoided overfitting issues. Moreover, this success is related to the two-layered neural network’s ability to effectively approximate nonlinear functions and reliably predict the PSS class value. Hence the ANN with two hidden layer is chosen to solve the PSS classification problem.

## 5. Performance Evaluation

In this section, the system sum rate and the spectral efficiency results from Monte Carlo simulations conducted in Matlab^©^ are presented to evaluate the performance of the proposed ANN-based PSS. The simulations were executed on a computer consisting of a Windows 10 64-bit operating system, Intel Core i7-8700 CPU, and 16 GB of RAM. Moreover, the impact of various system parameters, such as D2D bandwidth allocation and the UFBS transmit power $P_{u}$, on the performance of the proposed method is studied.

Furthermore, the proposed ANN-based PSS is compared against the standalone UFBS placement schemes k-medoids and k-means [7,9]. These two methods will be referred to as the k-means deployment process (MEA-DP) and the k-medoids deployment process (MED-DP). More specifically, comparisons are made between different networks schemes, such as the cooperative UAV-NOMA and D2D scheme termed as NOMA-D2D, and two standalone UAV transmission schemes without D2D communication capabilities between the GMTs, the UAV-NOMA optimal user pairing scheme [26], called NOMA, and the time domain UAV-OMA scheme, termed as OMA. In order to assess the performance of the proposed scheme as well as the compared ones, we define the spectral efficiency as:

(38) $$SE=\frac{R_{ach}}{B_{occ}},$$

where $R_{ach}$ is the achievable system sum rate and $B_{occ}$ denotes the total utilized network bandwidth. Concerning both the standalone OMA and NOMA transmission scheme, $B_{occ}=B_{u}$, while for the NOMA-D2D scheme, $B_{occ}=B_{d}+B_{u}$. The rest of the selected parameters regarding the abovementioned scenarios are listed in Table 5.

**Figure 7:**
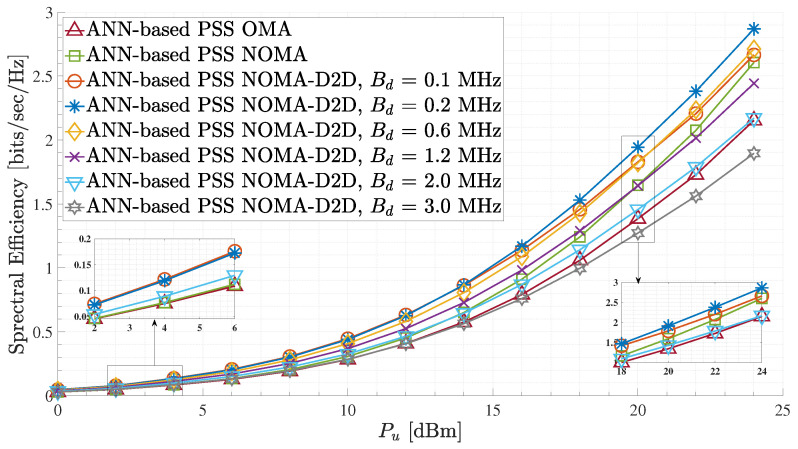
Spectral efficiency for the ANN-based PSS and different terrestrial D2D bandwidth values.

Figure 7 presents the spectral efficiency performance of the proposed ANN-based PSS for different terrestrial D2D bandwidth values and between the different network schemes. As it can be observed, the proposed ANN-based PSS scheme combined with the NOMA-D2D transmission technique for $B_{d}$ = 0.2 provides significant spectral efficiency gains compared to the other NOMA-D2D cooperative networks with $B_{d}\neq$ 0.2 and the standalone NOMA and OMA schemes. It is noteworthy that the proposed strategy, utilizing a $B_{d}$ equal to 0.1 MHz, exhibits comparable performance with a $B_{d}$ equals to 0.2 MHz for low UFBS transmit power values. Conversely, for high UFBS transmit power, the proposed strategy utilizing a $B_{d}$ equals to 0.2 MHz is determined to result in the near optimal spectral efficiency. Also, regarding the NOMA-D2D cooperative network with $B_{d}\leq$ 1.2 MHz, the proposed method achieves higher spectral efficiency gain than the standalone NOMA and the OMA scheme for all UFBS power transmission values. In contrast, for $B_{d}>$ 1.2 MHz, the suggested method’s spectral efficiency in a NOMA-D2D cooperative network is inferior to that of NOMA. This occurs because there is no need for additional bandwidth since the weak users’ rates are always constrained by the decoding rates of their signals at the strong users (21). Therefore, regarding the communication network, $B_{d}$ values greater than 1.2 MHz are considered a waste of resources. Additionally, for $B_{d}$ = 1.2 MHz, a switch case statement can be established. More specifically, in the case where the $P_{u}$ is lower than 20 dBm, the NOMA-D2D cooperative network outperforms the NOMA scheme, while for $P_{u}>20$ dBm, the standalone NOMA outperforms the NOMA-D2D cooperative scheme. This phenomenon occurs for large $P_{u}$ values since the A2G channel between the weak GMTs and the UFBS is strengthened, resulting in greater achievable rates for the weak GMTs via the direct A2G connection. Hence the D2D communication between the K pairs is mainly avoided, as the offered data rates via the D2D links are lower than those that can arise through the A2G links. This claim can be verified by expression in (21). Moreover, spectral efficiency degradation is observed when the terrestrial D2D bandwidth $B_{d}$ is greater than 1.2 MHz. In this case, the weak users can not efficiently exploit the capabilities offered by the wireless D2D channel link, as the rate received through the terrestrial cooperation is restricted by the decoding rates achieved by the strong users of each pair. This observation is derived as a result of the constraints imposed by (17)–(19), as well as from the explanation of cases 1 and 2 in Section 2.4. As an illustrative case for this phenomenon, the baseline standalone OMA scheme behaves better than the NOMA-D2D scheme with $B_{d}=3.0$ MHz in terms of spectral efficiency. Therefore, in the case of cooperative NOMA schemes such as the proposed one, the value of the terrestrial D2D bandwidth $B_{d}$ should be carefully chosen to avoid wasting spectrum resources. Also, in the NOMA-D2D cooperative network, for UFBS transmit power in the range of 0 to 12 dBm, it can be observed that the spectral efficiency is approximately the same for $B_{d}$ values equal to 0.1 and 0.2 MHz. However, for UFBS transmit power higher than 12 dBm, the proposed method with $B_{d}$ = 0.2 MHz achieves higher spectral efficiency than the others. In other words, $B_{d}$ = 0.2 MHz is a near-optimal D2D bandwidth value for the considered communication system.

**Figure 8:**
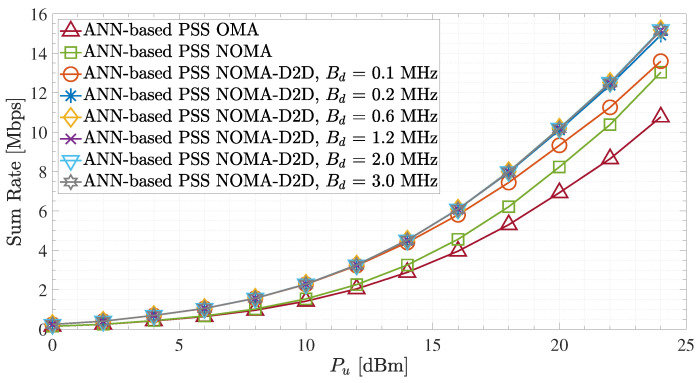
Sum rate for the ANN-based PSS and different terrestrial D2D bandwidth values.

In Figure 8, the sum rate performance of the proposed ANN-based PSS is examined for the different network schemes. It can be easily observed that the employment of the suggested PSS technique in the NOMA-D2D cooperative network readily outperforms OMA and NOMA schemes for all UFBS transmit power values and regardless of the D2D bandwidths value allocations. Moreover, for the NOMA-D2D cooperative network, we observe that the sum rate is approximately the same for any value of $B_{d}$ > 0.1 MHz. This can be supported by (21), which demonstrates that there is no need to devote more bandwidth to D2D transmission. Also, for UFBS transmit power in the range of 0 to 12 dBm, it can be observed that the sum rate is approximately the same for all $B_{d}$ values. Hence, large $B_{d}$ values for low-to-medium UFBS transmit powers are thus seen as a waste of resources. Therefore, for that UFBS transmit power range, there is a maximum value $B_{d}$, which should not be exceeded to avoid wasting resources. Nevertheless, the findings from Figure 7 and Figure 8 demonstrate that dynamic bandwidth allocation is required for D2D out-band communication to improve both the sum rate and spectral efficiency performance.

**Figure 9:**
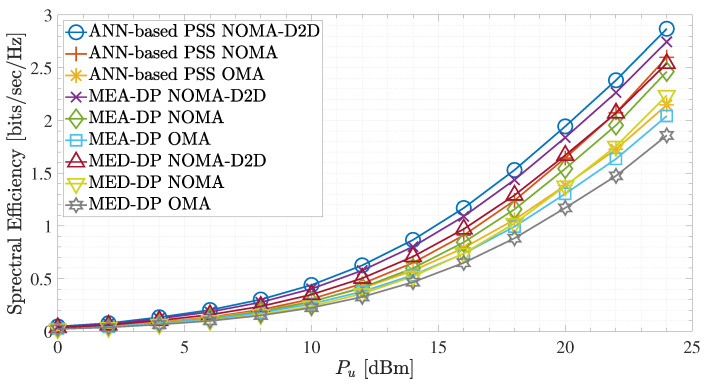
Spectral efficiency for $B_{d}$ = 0.2 MHz and different UFBS placement schemes.

**Figure 10:**
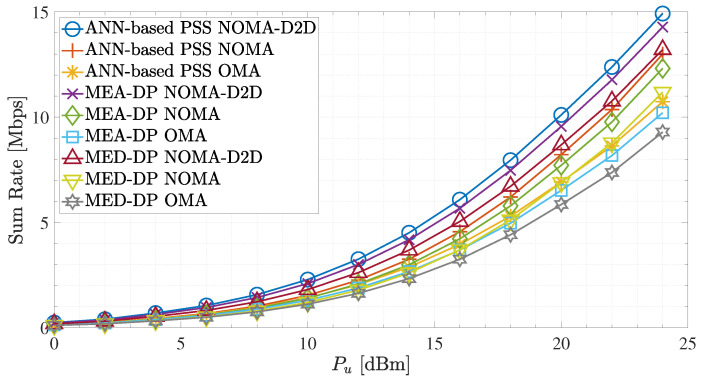
Sum rate for $B_{d}$ = 0.2 MHz and different UFBS placement schemes.

Figure 9 and Figure 10 show the effects caused by the different placement methods on the spectral efficiency and the system sum rate, respectively. More specifically, Figure 9 illustrates the spectral efficiency performance of the different communication schemes, NOMA-D2D with $B_{d}$ = 0.2 MHz, NOMA, and OMA, utilizing the different placement procedures. As can be observed, the ANN-based PSS applied to the NOMA-D2D cooperative network scheme achieves significant spectral efficiency gains compared to MEA-DP and MED-DP for all UFBS power transmission values. Also, observing all the network schemes individually (i.e., NOMA-D2D, NOMA, and OMA), the proposed ANN-based PSS outperforms the other two methods for all UFBS power transmission values. This results from the ability of the ANN to recognize patterns, indicating when each method should be conducted. Furthermore, regardless of the placement method, the cooperation between the aerial and D2D networks is promoted, i.e., the NOMA-D2D method, since it achieves the maximum spectral efficiency rates compared to standalone NOMA and OMA schemes. Moreover, for all UFBS transmission power values, the MEA-DP outperforms the MED-DP scheme in all three network configurations. This is justified by the explanation given in Section 4. Specifically, as the GMTs are placed randomly and uniformly in the region of interest, the probability of an outlying user appearing is very low. Consequently, in most cases, the k-means algorithm places the UFBS at such a point that it is equidistant by the users, thus improving the quality of channels gains against k-medoids. Lastly, the spectral efficiency of the ANN-based PSS applied to the standalone NOMA scheme is higher than that of MED-DP in the NOMA-D2D cooperative network scheme for $P_{u}$ values of approximately up to 22 dBm. This phenomenon occurs due to the improvement of the A2G channels through the proposed placement scheme. Consequently, in contrast to other cooperative systems, such as satellite D2D cooperative networks [27], the success of aerial and D2D cooperative networks strongly relies on the UFBS placement procedure. Hence, an inaccurate prediction concerning UAV’s position might degrade the overall network quality and lay the D2D network unnecessary.

Next, Figure 10 presents the sum rate for $B_{d}$ = 0.2 MHz and different placement procedures for NOMA-D2D, NOMA, and OMA network schemes. Throughout the $P_{u}$ range and regardless of the placement method scheme, it can be shown that the sum rate of the NOMA-D2D cooperative network is superior to that of NOMA and OMA, respectively. Similarly, as in spectral efficiency in Figure 9, the proposed ANN-based PSS outperforms the other two placement procedures for all network schemes. Moreover, it is observed that the proposed method, when applied in a NOMA scheme, can achieve higher spectral efficiency gains for the MED-DP applied in NOMA-D2D for $P_{u}$ > 22 dBm. Therefore, in such a scenario, with the deployment of the proposed method, we could avoid D2D transmission and save the entire D2D bandwidth.

Overall, the sum rate results of the NOMA-D2D cooperative scheme in all placement procedures indicate that the weak user’s achievable rate can be significantly improved. This advantage results from strong users cooperating with weak users of the system through out-band D2D communication.However, the sum rate and the spectral efficiency in all network schemes are heavily contingent on the UFBS placement within the region of interest. Regarding the results in Figure 7, Figure 8, Figure 9 and Figure 10, the proposed ANN-based PSS outperforms the other two methods in all network schemes and can offer terrestrial users reliable and high-quality communication.

Finally, Table 6 summarizes the key characteristics of the proposed ANN-based PSS and the compared MEA-DP and MED-DP schemes. Specifically, our method is less sensitive to outliers compared to MEA-DP, making it more robust in noisy environments. It also has higher reliability compared to both MEA-DP and MED-DP. Regarding spectral efficiency and sum rate, our method outperforms both MEA-DP and MED-DP, indicating that it may be a better choice for optimizing the utilization of resources and achieving higher data transmission rates in the given scenario.

## 6. Conclusions and Future Directions

Summarizing this paper, we proposed an ANN-based PSS method that maximizes the spectral efficiency and the sum rate in a NOMA-D2D cooperative network. It is the first time supervised ML methods are combined with unsupervised ones to enhance the placement procedure of the UFBS; the examples demonstrate the improvements achieved. To evaluate the performance of the ANN-based PSS policy, we compared it with two stand-alone unsupervised ML methods schemes. The results showed that the proposed method outperforms the other two in different network scenarios, such as NOMA-D2D cooperative, NOMA, and OMA schemes, regarding sum rate and spectral efficiency terms. Furthermore, the results show that utilizing the proposed method in a UAV-aided D2D-NOMA-cooperative network can offer terrestrial users reliable and high-quality communication compared with stand-alone NOMA or OMA schemes.

Possible future directions include studying various machine learning models as base learners and forming ensemble approaches to enhance the predictability of the placement procedure. Furthermore, in future work, we consider examining machine learning methods to identify the optimal D2D bandwidth value that achieves the maximum sum rate and, simultaneously, the maximum spectral efficiency regarding a UAV-aided D2D-NOMA-cooperative network. Finally, of potential interest is the integration of virtual MIMO in the context of aerial–terrestrial networks to improve communication between UAVs and other devices. Specifically, UAVs typically have limited size, weight, and power constraints, which can make it challenging to install multiple antennas and radio resources on them. By using virtual MIMO, various UAVs can work together as a single MIMO system and share their antennas and radio resources, increasing the range and capacity of the communication [41,42]. In addition, virtual MIMO can also improve the robustness of communication in UAV networks, as it can reduce the impact of fading and interference caused by the dynamic and often hostile environment in which UAVs operate.

## Figures and Tables

**Table 1:** System model parameters definition.

Parameter	Definition
*A*	Circle region of interest
$u_{l}$	3D location of each ${\mathrm{GMT}}_{l}$
$p_{1}$	3D location of UFBS
*N*	Total number of GMTs
*K*	Total number of GMT pairs
$G_{t}^{u}$	UFBS transmit antenna gain
$B_{u}$	UFBS bandwidth.
$P_{u}$	UFBS transmit power
$F_{u}$	UFBS operating frequency
$B_{d}$	D2D bandwidth
$P_{d}$	D2D transmit power
$F_{d}$	D2D operating frequency
$G_{t}^{d}$	D2D transmit antenna gain
$G_{r}^{d}$	D2D receive antenna gain
$N_{u}$	A2G receivers noise power
$N_{d}$	D2D receivers noise power
${\mathrm{PL}}_{l}^{u}$	A2G path loss for each ${\mathrm{GMT}}_{l}\;\left(1\leq l\leq N\right)$
${\mathrm{PL}}_{k}^{d}$	D2D path loss for each k pair of users $\left(1\leq k\leq K\right)$
$x_{k}^{u}$	Superimposed NOMA signal of each k pair∼(1 k K)
$\alpha_{i}$	Power allocation factor of the strong ${\mathrm{GMT}}_{i}$
$\Gamma_{l}^{u}$	A2G channel gain for each ${\mathrm{GMT}}_{l}\;\left(1\leq l\leq N\right)$
$y_{i}^{u}$	The received signal by the strong ${\mathrm{GMT}}_{i}$ from the UFBS
$y_{j}^{u}$	The received signal by the weak ${\mathrm{GMT}}_{j}$ from the UFBS
$y_{j}^{d}$	The received signal by the weak ${\mathrm{GMT}}_{j}$ from his pair’s strong ${\mathrm{GMT}}_{i}$ when the D2D cooperative transmission is activated.
$\gamma_{i}^{u}$	SNR of the A2G link of the strong ${\mathrm{GMT}}_{i}$ assuming perfect SIC
$\gamma_{k}^{u}$	SINR of the A2G link of the strong ${\mathrm{GMT}}_{i}$ for detecting the signal $s_{j}$ from his pair’s weak ${\mathrm{GMT}}_{j}$
$\gamma_{j}^{u}$	SINR of the A2G link of the strong ${\mathrm{GMT}}_{i}$ for detecting its own signal
$\gamma_{k}^{d}$	SINR of the D2D link of the weak ${\mathrm{GMT}}_{j}$ for detecting its own signal, which is relayed by his pair’s strong ${\mathrm{GMT}}_{i}$
$\Phi_{k}$	D2D channel gain for the weak ${\mathrm{GMT}}_{j}$ when the D2D cooperative transmission is activated
$R_{i}^{u}$	Maximum downlink NOMA achievable rate which succeed by the strong ${\mathrm{GMT}}_{i}$ through the A2G channel
$R_{j}^{u}$	Maximum downlink NOMA achievable rate which succeed by the weak ${\mathrm{GMT}}_{j}$ through the A2G channel
$R_{k}^{u}$	Maximum downlink NOMA achievable rate of the weak ${\mathrm{GMT}}_{j}$’s signal which succeed by his pair’s strong ${\mathrm{GMT}}_{i}$ through the A2G channel
$R_{k}^{d}$	Maximum achievable rate which succeed by the weak ${\mathrm{GMT}}_{j}$ through the D2D channel

**Table 2:** The features utilized in the developed ANN model.

Feature	Description
$D_{\mathrm{ptc}}$	The sum of distances between each ${\mathrm{GMT}}_{l}$ $\left(1\leq l\leq N\right)$ and the UFBS located in the centroid point $p_{1}^{c}\in A$
$D_{\mathrm{ptm}}$	The sum of distances between each ${\mathrm{GMT}}_{l}$ $\left(1\leq l\leq N\right)$ and the UFBS located in the medoid point $p_{1}^{m}\in A$
$r_{\min}^{p_{1}^{c}}$	The minimum 2D distance of the GMTs from the point $p_{1}^{c}\in A$
$r_{\max}^{p_{1}^{c}}$	The maximum 2D distance of the GMTs from the point $p_{1}^{c}\in A$
$r_{\min}^{p_{m}}$	The minimum 2D distance of the GMTs from the point $p_{1}^{m}\in A$
$r_{\max}^{p_{m}}$	The maximum 2D distance of the GMTs from the point $p_{1}^{m}\in A$
${\mathrm{PL}}_{\mathrm{sum}}^{p_{c}}$	The sum of the propagation losses of the GMTs in the case of placing the UFBS at the point $p_{1}^{c}\in A$
${\mathrm{PL}}_{\mathrm{sum}}^{p_{m}}$	The sum of the propagation losses of the GMTs in the case of placing the UFBS at the point $p_{1}^{m}\in A$

**Table 3:** Examined ANN layouts.

$l_{h}$	$m_{h}$	Layout	Converged Epoch	Minimum Loss Score	Training Time (s)
1	63	${\mathrm{ANN}}_{8-63-2}$	26	0.09	0.81
2	32/31	${\mathrm{ANN}}_{8-32-31-2}$	37	0.06	0.92
3	21/21/21	${\mathrm{ANN}}_{8-21-21-21-2}$	56	0.08	1.4
4	16/16/16/15	${\mathrm{ANN}}_{8-16-16-16-15-2}$	58	0.15	1.6

**Table 4:** Chosen hyperparameters values for ANNs models.

Parameters	Values
Activation functions	ReLU and SoftMax
Training algorithm	Gradient Descent
Learning rate	0.01
Maximum number of epochs to train	1000
Loss function	cross-entropy
Minimum performance gradient	$10^{-6}$

**Table 5:** Simulation parameters.

Parameters	Values
Simulated frames	100,000
Number of GMTs *N*	20
Region of interest circle radius *R*	500 m
UFBS downlink frequency $F_{u}$	1.8 GHz
D2D operating frequency $F_{d}$	2 GHz
UFBS transmit power $P_{u}$	0–24 dBm
GMT transmit power $P_{d}$	24 dBm
UFBS Tx antenna gain $G_{t}^{u}$	0 dBi
GMT Rx antenna gain $G_{r}^{g}$ with g={u,d}	0 dBi
GMT Tx antenna gain $G_{t}^{d}$	0 dBi
Terrestrial environment	Urban
Urban environment parameters	$a=9.61,b=0.16,\eta_{LoS}=1,\eta_{NLoS}=20$
UFBS bandwidth $B_{u}$	5 MHz
Receiver noise temperature $T_{g}$ with g={u,d}	24.6 dBK

**Table 6:** Comparison of the main properties of ANN-based PSS and MEA-DP and MED-DP schemes.

Performance Indicator	ANN-Based PSS	MEA-DP	MED-DP
Sensitive to outliers	No	Yes	No
Reliability	High	Medium	Medium
Spectral efficiency	High	Medium	Medium
Sum rate	High	Medium	Medium
Influenced by the distribution of GMTs in *A*	Medium	High	Medium
Fairness	High	High	Low

## Data Availability

The data that support the findings of this study are available from the corresponding author upon reasonable request.

## Abbreviations

The following abbreviations are used in this manuscript:

5G	Fifth Generation
6G	Sixth Generation
A2G	Air-to-Ground
ANN	Artificial Neural Network
AWGN	Additive White Gaussian Noise
B5G	Beyond 5G
D2D	Device-to-Device
DDPG	Deep Deterministic Policy Gradient
eICIC	Enhanced Inter-Cell Interference Coordination
FD	Full-Duplex
FSL	Free Space Pathloss
GD	Gradient Descent
GMT	Ground Mobile Terminal
HetNet	Heterogeneous Network
IoT	Internet of Things
KPI	Key Performance Indicator
LoS	Line of Sight
LTE	Long Term Evolution
MDQN	Mutual Deep Q-Network
MEA-DP	k-Means Deployment Process
MED-DP	k-Medoids Deployment Process
MIMO	Multiple-Input Multiple Output
ML	Machine Learning
MLP	Multi-Layer Perceptron
NLoS	Non Line of Sight
NOMA	Non-Orthogonal Multiple Access
OMA	Orthogonal Multiple Access
PSS	Placement Scheme Selection
QoS	Quality-of-Service
QSM	Quadrature Spatial Modulation
RF	Radio Frequency
SC	Superposition Coding
SIC	Successive Interference Cancellation
TBS	Terrestrial Base station
UAV	Unmanned Aerial Vehicle
UFBS	UAV Flying Base Station
